# Unveiling the influence of adaptation time on xylanase and arabinoxylan-oligosaccharide efficacy: a study on nutrient digestibility, viscosity, and scanning electron microscopy in the small and large intestine of growing pigs fed insoluble fiber

**DOI:** 10.1093/jas/skad378

**Published:** 2023-11-22

**Authors:** Amy L Petry, Nichole F Huntley, Michael R Bedford, John F Patience

**Affiliations:** Department of Animal Science, Iowa State University, Ames, IA 50011, USA; Department of Animal Science, Iowa State University, Ames, IA 50011, USA; AB Vista Feed Ingredients, Marlborough, Wiltshire SN8 4AN, United Kingdom; Department of Animal Science, Iowa State University, Ames, IA 50011, USA

**Keywords:** adaptation time, corn fiber, ileal digestibility, jejunal digestibility, swine, xylanase

## Abstract

The experiment objective was to evaluate the impact of xylanase over time on viscosity and digestibility in growing pigs fed corn-based fiber. Twenty gilts with an initial body weight of 30.6 ± 0.2 kg (*n* = 5 per dietary treatment) were fitted with t-cannulae in the medial jejunum and terminal ileum, housed individually, and randomly assigned to one of four dietary treatments: low-fiber control (**LF**) with 10.4% total dietary fiber (**TDF**), 30% corn bran high-fiber control (**HF**; 26.4% TDF), HF + 100 mg xylanase/kg (**XY**; Econase XT 25P; AB Vista, Marlborough, UK), and HF + 50 mg arabinoxylan-oligosaccharide/kg (**AX**). Gilts were limit fed for three 17 d periods (**P1, P2, P3**); each included 5 d adaptation, 2 d fecal collection, 3 d ileal collection, 3 d jejunal collection, and 4 d related rate of passage study. Data were analyzed as repeated measures using a linear mixed model with surgery date as a random effect, and dietary treatment, period, and their interaction as fixed effects. Jejunal and ileal digesta viscosity did not differ among dietary treatments or periods (*P* > 0.10). There was a dietary treatment × period interaction for the apparent jejunal digestibility (**AJD**) of dry matter (**DM**), gross energy (**GE**), insoluble dietary fiber (**IDF**), neutral detergent fiber (**NDF**), total arabinoxylan (**T-AX**), total non-starch polysaccharide (**T-NSP**), and TDF (*P*≤ 0.05). In P1, LF had the greatest AJD of DM (15.5%), and relative to HF and AX, XY decreased it (9.3%, 10.1 %, and 6.3%, respectively). In P2, the AJD of DM in XY was greater than HF (11.7% vs. 9.1%) but did not differ from AX (10.5%). Relative to HF, in P3, XY increased AJD of DM (11.7 vs 15.3%), and AX decreased it (7.2%). For the AJD of NDF, AX performed intermediately in P1; in P2, relative to HF, XY, and AX increased the AJD of NDF (8.4%, 13.1%, and 11.7%, respectively), and in P3, XY, and LF did not differ (13.6 vs. 14.4%). A similar response was observed for the AJD of IDF and TDF, except for XY having the greatest AJD of IDF, T-AX, T-NSP, and TDF in P3 (*P* < 0.05). Compared to LF, irrespective of period, HF decreased the apparent ileal digestibility (**AID**) and apparent total tract digestibility (**ATTD**) of IDF, TDF, and NDF (*P* < 0.05). Relative to HF, XY partially mitigated this effect, improving the AID and ATTD of TDF, IDF, and NDF (*P* < 0.05). Increased corn-based fiber decreased nutrient digestibility, but XY partially mitigated that effect in the small intestine through enhanced fiber digestibility when given sufficient adaptation time.

## Teaser

This study aimed to investigate the impact of adaptation time on the effectiveness of xylanase in improving nutrient and energy digestibility, as well as reducing digesta viscosity in growing pigs fed a high-fiber corn-based diet. Study findings indicate xylanase can partially mitigate the impact of corn-based fiber on nutrient digestibility by enhancing arabinoxylan digestibility in the jejunum when given sufficient adaptation time. This discovery sheds light on the time required for pigs to respond to xylanase supplementation in corn-based diets.

## Introduction

Recently, there has been an increase in both research and the use of exogenous enzymes within the swine industry. This increased interest in exogenous enzymes has been stimulated by the success of phytase in monogastric nutrition, and carbohydrase usage within the poultry industry. Xylanase is a carbohydrase that facilitates the hydrolysis of arabinoxylans and has the potential to improve the utilization of fiber by the pig. Many swine diets across the globe contain substantial levels of arabinoxylans originating from the cereal grains and industrial co-products within the diet (Jaworski et al., 2015). Improving the utilization of arabinoxylans from corn may increase the contribution of energy from fiber and mitigate its antinutritive effects, both of which would reduce the cost of pork production (Abelilla and Stein, 2019; Patience and Petry, 2019). The role of xylanase in mitigating increased intestinal viscosity and improving nutrient digestibility in poultry is well-established (Raza et al., 2019). This positive impact is largely due to the enzymatic breakdown of arabinoxylans improving the emulsification of nutrients within the digesta milieu (Dikeman and Fahey, 2006). However, unlike in poultry, the efficacy of xylanase in swine production is highly variable, particularly when supplemented in corn-based diets. A recent review of the literature concluded that xylanase supplementation in corn-based diets was more successful at increasing fiber digestibility than it was at improving pig growth performance (Torres-Pitarch et al., 2019; Petry and Patience, 2020).

These inconsistencies in digestibility and performance responses have raised questions about the mechanism of action of xylanase, and under what conditions is it most effective in improving the utilization of fiber. Due to the variability in experimental conditions and designs within the current body of literature, it is difficult to isolate under what conditions xylanase is most effective (Adeola and Cowieson, 2011). However, adaptation time may be one factor that impacts the efficacy of xylanase in swine production. A study by Petry et al. (2020a) found xylanase improved the average daily gain and feed efficiency of pigs fed a high-fiber corn-based diet, but it required between 14 and 27 d to observe a response. Moreover, research by Huntley and Patience (2018), observed that xylose retention, a potential release product of xylanase, increased with greater adaption time. Adaptation time may also impact the efficacy of xylanase to improve the total tract digestibility of nutrients in growing pigs fed corn-based diets (Weiland and Patience, 2021), and growth performance of nursery pigs (Moita et al., 2022). However, there is a paucity of studies directly investigating the impact of adaptation time on the efficacy of xylanase across the pig’s gastrointestinal tract.

Recently, the stimbiotic potential of xylanase-release products has been proposed in pigs (González-Ortiz et al., 2019). One of the primary potential release products of xylanase is arabino-xylooligosaccharides (**AXOS**; Kouzounis et al., 2022). In poultry, supplementing AXOS, instead of xylanase, has been shown to increase fiber digestibility through a stimbiotic mechanism by stimulating a more metabolically active microbiome (Bautil et al., 2020). However, it is unclear if AXOS supplementation effects are translatable to pigs and the role of adaptation time. Collectively, the objective of this experiment was to determine if xylanase or AXOS would progressively reduce viscosity and improve nutrient and energy digestibility over time in multiple gastrointestinal locations of growing pigs fed a diet higher in corn-based fiber. It was hypothesized that the efficacy of xylanase and AXOS to improve nutrient and energy digestibility, and attenuate digesta viscosity, would improve with increasing adaptation time.

## Materials and Methods

All experimental procedures were approved by the Iowa State University Institutional Animal Care and Use Committee (#2-18-8705-S). This experiment adhered to guidelines for the ethical and humane use of animals for research according to the Guide for the Care and Use of Agricultural Animals in Research and Teaching (FASS, 2010).

### Animals, Housing, and Experimental Design

Twenty crossbred growing gilts (progeny of Camborough sows × 337 sires; PIC Inc., Hendersonville, TN) were used for this experiment. They were surgically fitted with simple t-cannulae in the terminal ileum and the medial jejunum. Pigs underwent the surgical procedure described by Petry et al. (2020b); the ileal cannula was placed approximately 10 cm cranial to the ileocecal valve and a second t-cannula in the jejunum 240 cm distal from where the duodenum is visually posterior to the transverse colon as identified by the Treitz ligament. Gilts were housed in individual pens (1.2 × 1.5 m) equipped with a nipple waterer, and a half-slatted concrete floor in an environmentally controlled facility. After recovery from surgery, pigs were weighed (30.6 ± 0.2 kg), blocked by d of surgical procedure, and randomly allotted to one of four dietary treatments: a low-fiber control (**LF**) with 10.4% total dietary fiber (**TDF**), a 30% corn bran high-fiber control (**HF**; 26.4% TDF), HF + 100 mg xylanase/kg (**XY**; Econase XT 25P; AB Vista, Marlborough, UK), and HF + 50 mg arabinoxylan-oligosaccharide/kg (**AX**; 3 - 7 degrees of polymerization).

Corn bran without solubles was added to the LF dietary treatment at the expense of corn for high-fiber dietary treatments (HF, XY, and AX; Table 1). The assayed composition of the dietary treatments, with particular emphasis on fiber components, is presented in Table 2. Similarly, xylanase or the arabinoxylan-oligosaccharide was added at the expense of corn in the XY and AX dietary treatments, respectively. All other ingredients remained constant, and thus, nutrients were allowed to float. This approach minimizes the differences in diet composition among dietary treatments and reduces the risk of confounding experimental outcomes. Concentrations of amino acids, vitamins, and minerals met or exceeded their estimated requirements (NRC, 2012). Titanium dioxide (TiO_2_) was added at 0.4% as an indigestible marker. All pigs were provided with the same daily amount of feed equivalent to 3.2 times the maintenance ME requirement based on the average of the ME content among dietary treatments (NRC, 2012) and the average BW of the pigs. Daily feed allowance was divided into two equal meals given at 0730 and 1630 hours. All diets were manufactured in mash form, and pigs had ad libitum access to water. Gilts were limit-fed for three 17 d periods (**P1**, **P2**, or **P3**). Each period included 5 d of adaptation, 2 d of fecal collections, 3 d of ileal collections, 3 d of jejunal collections, and 4 d of rate of passage collections for a related study. At the end of each collection period, all pigs were weighed, and the daily feed allowance for the next collection period was adjusted. Gilts remained on the same dietary treatment for the full course of the experiment to characterize the impact of dietary treatment over time.

**Table 1.** Ingredient and nutrient composition of dietary treatments (as-fed basis)^1^

	Dietary treatment			
Item	LF	HF	XY	AX
Ingredient composition, %				
Corn	74.035	44.036	44.026	44.031
Corn bran without solubles	0.000	30.000	30.000	30.000
Soybean meal	22.347	22.347	22.347	22.347
Limestone	1.231	1.231	1.231	1.231
Monocalcium phosphate 21%	0.569	0.569	0.569	0.569
Salt	0.500	0.500	0.500	0.500
TiO_2_	0.400	0.400	0.400	0.400
L-lysine HCL	0.322	0.322	0.322	0.322
Trace mineral premix^2^	0.200	0.200	0.200	0.200
Vitamin premix^3^	0.250	0.250	0.250	0.250
L-threonine	0.078	0.078	0.078	0.078
DL-methionine	0.063	0.063	0.063	0.063
Quantum Blue, 5G	0.005	0.005	0.005	0.005
Econase 25 P	0.000	0.000	0.010	0.000
Arabinoxylan-oligosaccharide^4^	0.000	0.000	0.000	0.005
Calculated nutrients				
SID^5^ Lysine, %	0.98	1.00	1.00	1.00
SID TSAA^6^:Lysine	0.56	0.56	0.56	0.56
SID Threonine: Lysine	0.60	0.60	0.60	0.60
SID Trpytophan:Lysine	0.17	0.17	0.17	0.17
Ca, %	0.66	0.65	0.65	0.65
STTD^7^ P, %	0.33	0.31	0.31	0.31
ME, Mcal/kg	3.25	3.01	3.01	3.01
NE, Mcal/kg	2.45	2.24	2.24	2.24

^1^Dietary treatments include a low-fiber control (LF) = basal corn-soybean diet; high-fiber control (HF) = basal corn-soybean diet with 30% corn bran at the expense of corn; HF with the addition of xylanase (XY); HF with the addition of arabinoxylan-oligosaccharide (AX).

^2^Trace mineral premix provided the following (per kilogram diet): 165 mg of Fe (ferrous sulfate); 165 mg of Zn (zinc sulfate); 39 mg of Mn (manganese sulfate); 16.5 mg of Cu (copper sulfate); 0.3 mg of I (calcium iodate); 0.3 mg of Se (sodium selenite).

^3^Vitamin premix provided the following (per kilogram diet): 6,125 IU of vitamin A; 700 IU of vitamin D3; 50 IU of vitamin E; 3 mg of menadione (to provide vitamin K); 11 mg of riboflavin; 27 mg of d-pantothenic acid; 0.05 mg of vitamin B12, and 56 mg of niacin.

^4^3 to 7° of polymerization.

^5^Standard ileal digestible.

^6^Total sulfur amino acids (Met + Cys).

^7^Standardized total tract digestible.

**Table 2.** Analyzed composition of dietary treatments (as-fed basis)^1^

	Dietary treatment			
Item	LF	HF	XY	AX
Analyzed composition, %				
Dry matter	88.1	86.2	86.8	87.1
Crude protein	15.3	14.8	14.7	14.8
Acid-hydrolyzed ether extract	3.3	2.8	2.6	2.6
Titanium dioxide	0.39	0.40	0.39	0.40
Total NSP	7.1	19.7	19.8	20.2
Insoluble NSP	6.4	18.9	18.7	19.1
Soluble NSP	0.7	0.8	0.8	1.1
Total dietary fiber	10.4	26.4	27.1	27.0
Insoluble dietary fiber	9.8	25.4	26.0	25.9
Soluble dietary fiber	0.6	1.0	1.1	1.1
Neutral detergent fiber	7.3	23.4	23.5	23.4
Acid detergent fiber	2.4	5.6	5.8	5.9

^1^Dietary treatments include a low-fiber control (LF) = basal corn-soybean diet; high-fiber control (HF) = basal corn-soybean diet with 30% corn bran at the expense of corn; HF with the addition of xylanase (XY); HF with the addition of arabinoxylan-oligosaccharide (AX).

### Sample Collections

Diet samples were collected at the time of mixing, thoroughly homogenized, and carefully subsampled. Fresh fecal subsamples were obtained from each pig via grab sampling. Ileal samples were collected by attaching a 207-mL plastic bag (Whirl-Pak; Nasco, Fort Atkinson, WI) to the opened cannula with a cable tie. Bags were removed once they were filled with digesta or at least every 30 min for 8 h per collection day. A jejunal sample was collected once per hour for 6 h post feeding. All samples were immediately stored at –20 °C to avoid degradation. Prior to being assayed, fecal samples were thawed, homogenized, subsampled, and oven-dried in a convection oven at 60 °C until a constant weight was reached. Ileal and jejunal samples were thawed, homogenized, subsampled, and lyophilized. Diets and dried ileal, jejunal, and fecal subsamples were ground in a Wiley Mill (Variable Speed Digital ED-5 Wiley Mill; Thomas Scientific, Swedesboro, NJ) through a 1-mm screen and stored in desiccators to maintain a constant dry matter **(DM**) content.

### Chemical Analytical Methods

Feed, ileal, and fecal samples were analyzed in duplicate for acid-hydrolyzed ether extract (**AEE**; method 2003.06 of AOAC, 2007), and nitrogen (TruMac; LECO Corp., St. Joseph, MI; method 990.03 of AOAC, 2007). An ethylenediaminetetraacetate sample (9.56 ± 0.05% nitrogen) was used for standard calibration and crude protein (**CP**) was calculated as nitrogen × 6.25. Diet, jejunal digesta, ileal digesta, and fecal samples were analyzed in duplicate for DM (method 930.15 of AOAC, 2007), TiO_2_ (Leone, 1973), and gross energy (**GE**). An isoperibolic bomb calorimeter (model 6200; Parr Instrument Co., Moline, IL) was used to determine GE, and benzoic acid (6,318 kcal GE/kg; Parr Instrument Co.) was used as the standard for calibration and was determined to contain 6,321 ± 6 kcal GE/kg. Diet, jejunal digesta, ileal digesta, and fecal samples were analyzed for neutral detergent fiber (**NDF**; Van Soest and Robertson, 1979) and for acid detergent fiber (**ADF**; Goering and Van Soest, 1970). Insoluble dietary fiber (**IDF**) and soluble dietary fiber (**SDF**) were determined in tandem using the Ankom TDF Dietary Fiber Analyzer (AOAC 991.43; AOAC, 2007; Ankom Technology, Macedon, NY). The TDF content was calculated as the sum of IDF and SDF. Diet, jejunal digesta, and ileal digesta were analyzed in triplicate for total non-starch polysaccharides (**T-NSP**), total arabinoxylans (**T-AX**), total cellulose (**T-CEL**), insoluble non-starch polysaccharides (**I-NSP**), insoluble arabinoxylans (**I-AX**), and insoluble cellulose (**I-CEL**) using a gas chromatography mass spectrometer in electron impact mode (Agilent Technologies, Palo Alto, CA, USA) according to the methods and conditions described by Englyst et al. (1994). Myo-inositol was used as an internal standard. A coefficient of variation threshold of less than 1% was used for DM, TiO_2_, CP, and GE, less than 3% for NDF, ADF, and AEE, and 5% for IDF, SDF, TDF, T-NSP, T-AX, T-CEL, I-NSP, I-AX, and I-CEL.

### Viscosity

**Figure 1.**
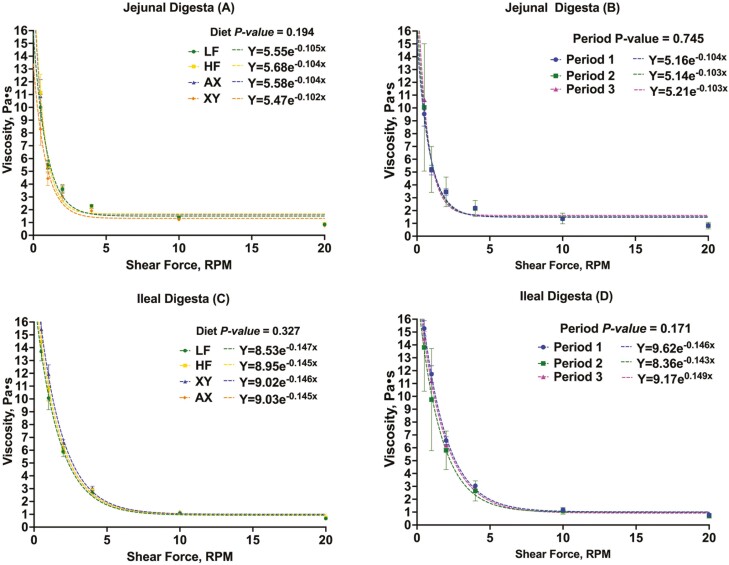
The relationship between viscosity measured at a given shear rate for jejunal and ileal digesta. (**A**) The impact of dietary treatment on the viscosity of jejunal digesta. (**B**) The effect of period on the viscosity of jejunal digesta. (**C**) The impact of dietary treatment on the viscosity of ileal digesta. (**D**) The effect of period on the viscosity of ileal digesta.

The viscosity of a pooled subsample of jejunal and ileal whole digesta was measured using a DV rotational viscometer equipped with a V3 vane mixing spindle (Brookfield, Middleboro, MA, USA). Concisely, samples were incubated in a water bath at 90 °C for 30 min to cease enzyme activity. Samples were then homogenized and cooled to 37 °C in a water bath. Approximately 30 mL of digesta was placed in 100 mL glass beakers with a diameter of 5.2 cm and measured at 0.5, 1, 2, 4, 10, and 20 rpm. The sample temperature was maintained at 37 °C ± 0.7 to simulate a physiologically relevant impact of temperature on viscosity. Due to the non-Newtonian shear-thinning nature of digesta, viscosity was expressed as the exponential relationship of the observed shear-thinning behavior in Pa**·**s (Figure 1).

### Scanning Electron Microscopy

Scanning electron microscopy (**SEM**) images depicting the topography of corn bran and jejunal and ileal digesta samples from HF, XY, and AX at each period were captured using field emission SEM at the Roy J. Carter High Resolution Microscopy Facility (Iowa State University, Ames, IA). Samples were uniformly mounted on circular aluminum stubs with double-sided carbon tape and coated with platinum to a maximum thickness of 8 nm using a high-resolution sputter coater adapted with a high-resolution thickness controller (HR 208, Cressington Scientific Instruments Ltd, Watford, UK). Samples were examined with an ultra-low voltage cold cathode field emission scanning electron microscope (Hitachi S-4800, Hitachi, Krefeld, Germany) at a voltage of 10.0 kV. The corresponding dietary treatment and period for a given sample were blinded to the technician imaging the samples. Multiple representative pictures were captured at 100x magnification, and images were scaled with an associated software (4800 FE-SEM Hitachi Internal Software, Hitachi; Krefeld, Germany). A representative picture of each dietary treatment for each period was selected by a research technician blinded to dietary treatments and experimental hypotheses to avoid confirmation bias in selecting a representative image.

### Calculations and Statistical Analysis

The apparent jejunal digestibility (**AJD**), apparent ileal digestibility (**AID**), and apparent total tract digestibility (**ATTD**) of nutrients and energy were calculated according to the equation of Oresanya et al. (2007).

Data were analyzed according to the following linear mixed model:


$$\begin{array}{l} Y_{ijkl}=\;\mu +\tau_{i\;}+\upsilon_{j}+\;\tau_{i\;}\upsilon_{j}+\rho_{k\;}+e_{ijkl} \end{array}$$

Where $Y_{ijkl}$ is the observed value for a given gilt within the *i*th level of dietary treatment and *j*th level of a period of the *k*th surgical replicate; μ is the general mean; $\tau_{i\;\;\;}$ is the fixed effect of the *i*th dietary treatment (*i* = 1 to 4); ${\;\upsilon \;}_{j}$ is the fixed effect of the *j*th period (*j* = 1 to 3); $\tau_{i\;}{\;\upsilon \;}_{j}$ is the interaction term of Dietary Treatment ×    Period; $\rho_{k\;\;\;}$ is the random effect of the *k*th surgical replicate (*k* = 1 to 6); and $e_{ijkl}$ is the associated variance as described by the model for $Y_{ijkl}$ (*l* = 1 through 5); assuming $\rho_{j}\;∼N\left(0,\;\sigma_{\rho }^{2}\right),\;\;\;\;\;$ and $e_{ijk}\;\;\;∼N(0,\;ARI\;\sigma_{e}^{2})$, where I is the identity matrix.

The PROC UNIVARIATE procedure in SAS 9.3 (SAS Inst., Cary, NC) was used to verify the normality and homogeneity of the studentized residuals. The aforementioned mixed model was analyzed using PROC MIXED. The best fit exponential function for the non-Newtonian shear-thinning relationship of jejunal and ileal viscosity was determined and analyzed using the *drc, nlme*, and *aomisc* packages of RStudio Version 1.2.503 (Free Software Foundation., Boston, MA). The best fit exponential function selected using BIC criteria was plotted alongside those data. Least square means were separated using Fisher’s Least Significant Difference test, and dietary treatment differences were considered significant if *P* ≤ 0.05 and trends if 0.05 > *P* ≤ 0.10. A weighted average of SEM among fixed effects was pooled and reported along with dependent variables.

## Results

No interactions were identified between surgical replicate and other main effects for the reported dependent variables; therefore, it was included as a random effect to adjust for additional variance. For a given variable, when the interaction term was significant, the simple effects of dietary treatment were presented. Otherwise, when the interaction term did not reach a level of significance, the main effects of dietary treatment were reported.

### Viscosity

The viscosity of jejunal digesta did not differ among dietary treatments (Fig. 1A; *P* = 0.194), nor did period impact jejunal viscosity (Fig. 1B; *P* = 0.745). Similarly, neither dietary treatment nor period impacted the viscosity of ileal digesta (Fig. 1C; *P* = 0.327 and Fig. 1D; *P* = 0.171, respectively). However, as expected, the viscosity of ileal digesta was numerically greater than jejunal digesta.

### Apparent Jejunal Digestibility

**Figure 2.**
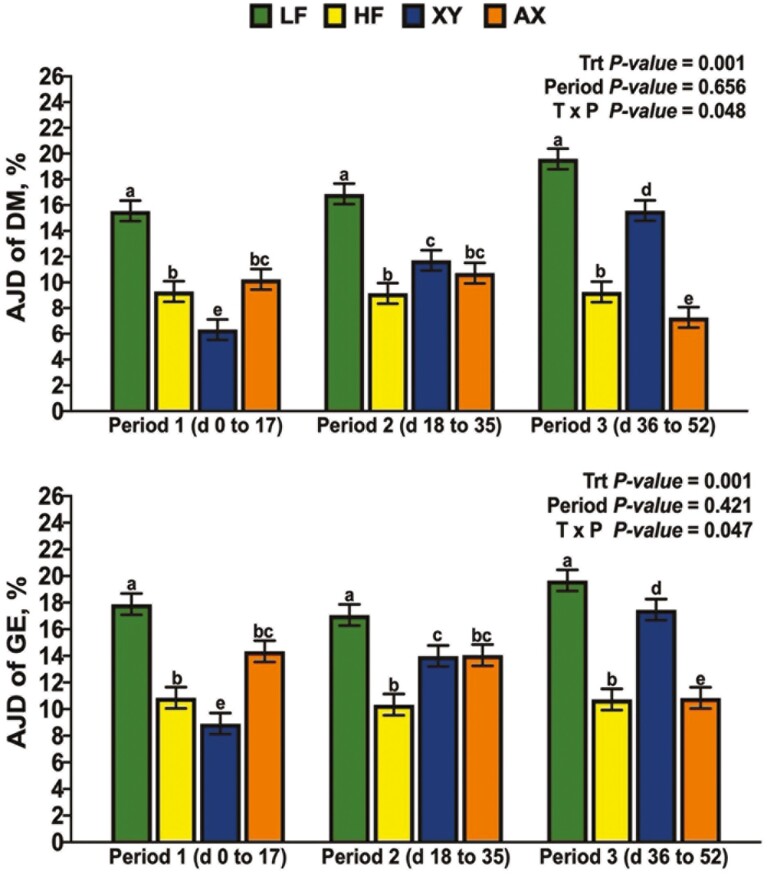
The simple effects of dietary treatment by period on the apparent jejunal digestibility (**AJD**) of dry matter (**DM**) and gross energy (**GE**).

**Figure 3.**
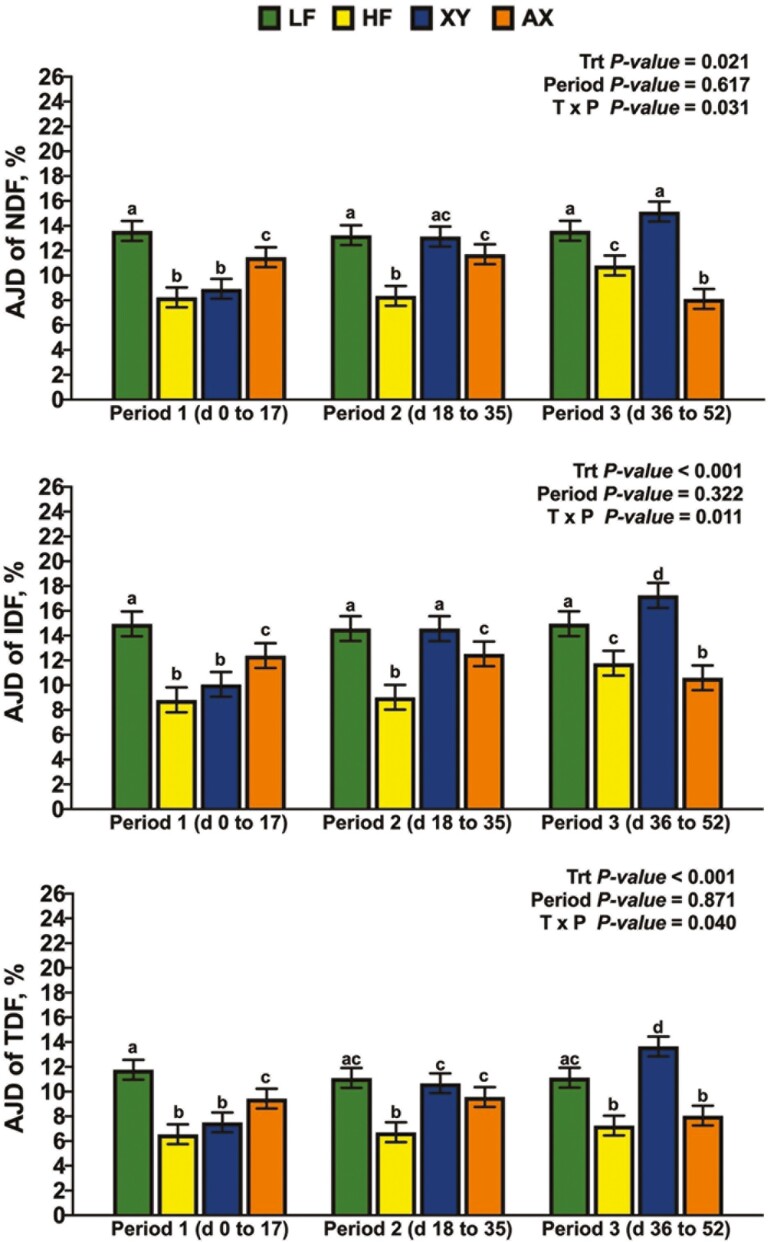
The simple effects of dietary treatment by period on the apparent jejunal digestibility (**AJD**) of neutral detergent fiber (**NDF**), insoluble dietary fiber (**IDF**), and total dietary fiber (**TDF**).

**Figure 4.**
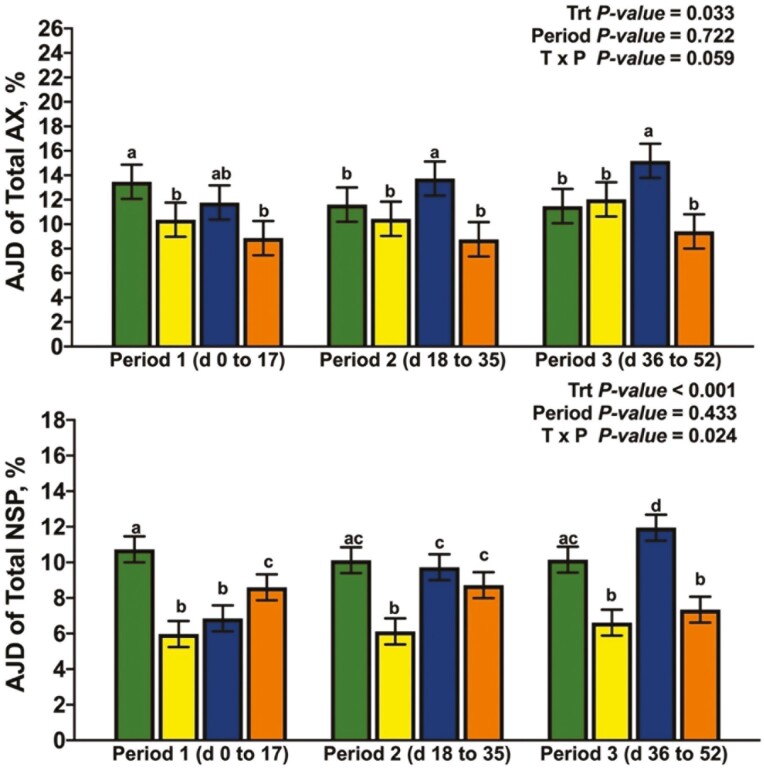
The simple effects of dietary treatment by period on the apparent jejunal digestibility (**AJD**) of total arabinoxylan-oligosaccharide (**T-AX**) and total non-starch polysaccharide (**T-NSP**).

**Figure 5.**
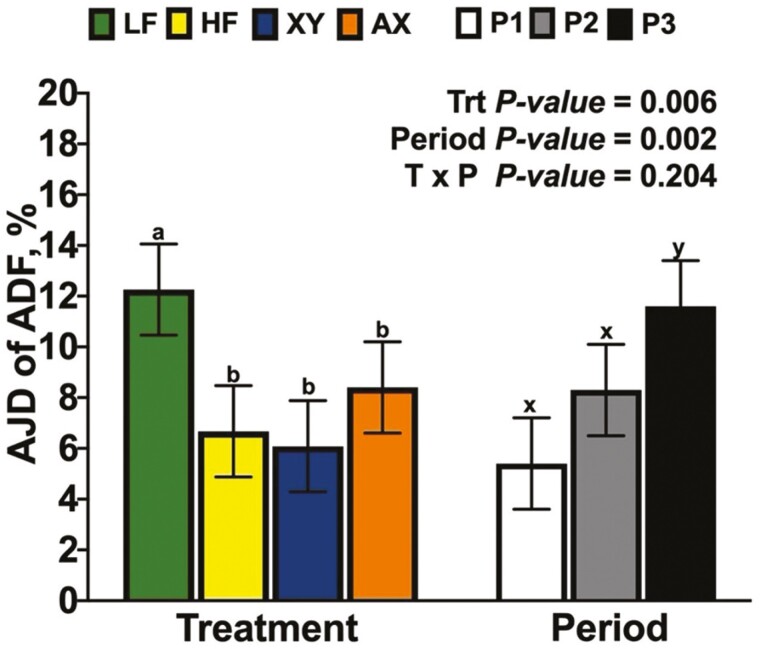
The main effects of dietary treatment and period for the apparent jejunal digestibility (**AJD**) of acid detergent fiber (**ADF**).

There was a significant interaction between dietary treatment and period for the AJD of DM and GE (Fig. 2; *P* = 0.048 and *P *= 0.047, respectively). The interaction between dietary treatment and period for the AJD of DM and GE was driven largely by the response of XY and AX; the AJD of DM and GE for XY were lowest among dietary treatments in P1, greater than HF, but not AX in P2, and XY had an intermediate response among dietary treatments in P3. In contrast, AX increased the AJD of DM and GE over XY in P1 but did not differ from HF and had the lowest AJD of DM among dietary treatments in P3. Likewise, the interaction term for dietary treatment by period was significant for the AJD of NDF, IDF, and TDF (Fig. 3; *P *= 0.031, *P *= 0.011, and *P* = 0.04, respectively). The response of XY and AX across periods drove the interaction for AJD of NDF in that AX was intermediate among dietary treatments and XY did not differ from HF in P1. Dietary treatment XY increased the AJD of NDF relative to HF but did not differ from LF or AX in P2; in P3, AX decreased the AJD of NDF relative to all other dietary treatments. A similar response across periods was observed for the AJD of IDF and TDF, except for XY improving the AJD of IDF and TDF in P3 above all dietary treatments (*P *< 0.05). There tended to be an interaction between dietary treatment and period for the AJD of T-AX (Fig. 4; *P* = 0.059), and a significant interaction for the AJD of T-NSP (*P* = 0.024). Supplementing xylanase increased AJD of T-NSP and T-AX in P2 and P3, but AX only influenced the P1 and P2 digestibility of T-NSP (*P *< 0.05). The interaction between dietary treatment and period was not significant for the AJD of ADF (Fig. 5; *P* = 0.204), but LF had the greatest AJD of ADF among dietary treatments (*P* = 0.006) and the AJD of ADF was greater in P3 than in P1 and P2 (*P* = 0.002).

### Apparent Ileal Digestibility

**Figure 6.**
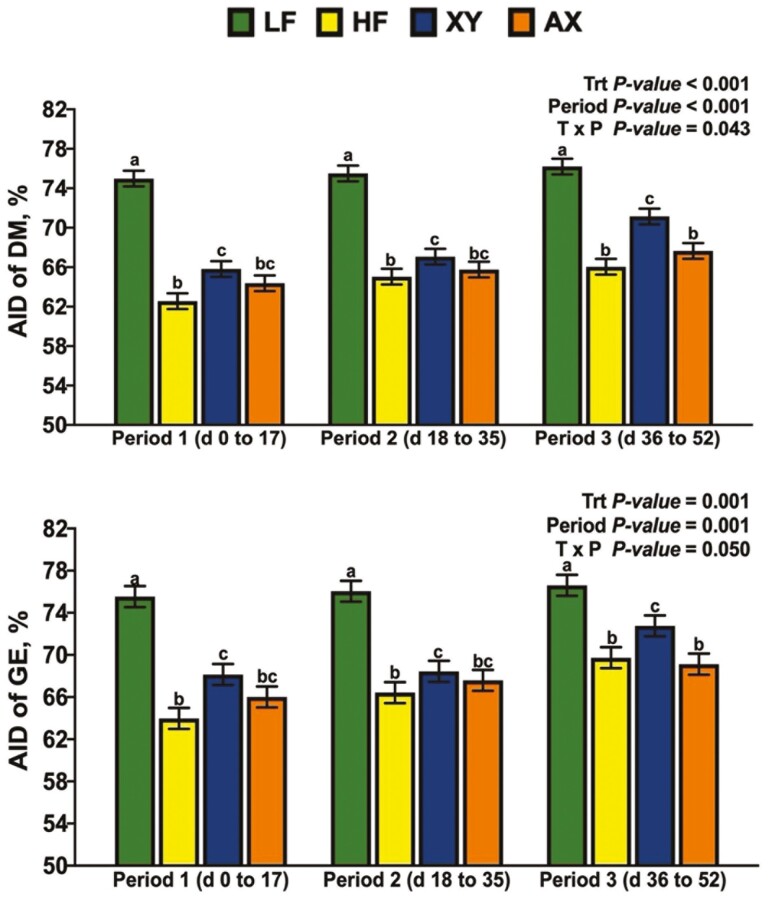
The simple effects of dietary treatment by period on the apparent ileal digestibility (**AID**) of dry matter (**DM**) and gross energy (**GE**).

There was a significant interaction between dietary treatment and period for the AID of DM and GE (Fig. 6; *P *= 0.043 and *P *= 0.05, respectively). The AID of DM and GE were greatest for LF but did not differ among periods. As expected, HF decreased the AID of DM and GE relative to LF, but the digestibility coefficients for HF did not differ among periods. In P1 and P2, the AID of DM and GE of XY was greater than HF, but not AX. However, in P3, XY increased the AID of DM and GE over both HF and AX. There was no significant interaction for dietary treatment by period for the AID of CP, AEE, TDF, IDF, NDF, and ADF (*P* > 0.10), and the main effects are presented in Table 3. Among dietary treatments, LF had the greatest AID of CP (*P *= 0.017), and the AID of CP did not differ among periods (*P *= 0.92). Similarly, LF had the greatest AID of AEE (*P *= 0.26), and the AID of AEE did not differ among periods (*P* = 0.348). Relative to LF, HF decreased the AID of TDF by 23.8%, and XY increased the AID of TDF by 21.7% relative to HF (*P *< 0.01). Among periods, the AID of TDF was greater in P2 and P3 than in P1 (*P *= 0.002). Compared to LF, HF reduced the AID of IDF by 21.9%, but relative to HF, XY and AX increased the AID of IDF by 20.4% and 15.3%, respectively (*P *< 0.001). Similarly, for the AID of NDF, relative to LF, HF decreased the AID of NDF, but XY and AX increased the AID of NDF, compared to HF (*P* < 0.004). The AID of IDF and NDF was greatest in P3 (*P *= 0.007 and *P *= 0.031, respectively). The AID of ADF did not differ among dietary treatments or periods (*P *> 0.20). Relative to HF, xylanase supplementation increased the AID of T-NSP, T-AX, I-NSP, and I-AX, irrespective of the period (Table 4; *P *< 0.05). Pigs fed LF had the greatest AID of T-CEL and I-CEL (*P *< 0.05). Across periods, the AID of T-NSP and I-NSP was greater in P3 than in P1 and P2 (*P *< 0.05).

**Table 3.** The main effects^1^ of dietary treatment and period on the AID of crude protein (**CP**), acid-hydrolyzed either extract (**AEE**), total dietary fiber (**TDF**), insoluble dietary fiber (**IDF**), neutral detergent fiber (**NDF**), and acid detergent fiber (**ADF**)

	Dietary treatment^2^				Period			SEM^3^	Trt^4^ *P-*value	Period *P-*value
Item	LF	HF	XY	AX	1	2	3			
AID^5^, %										
CP	79.4^a^	76.6^b^	77.1^b^	76.4^b^	77.3	77.5	77.4	0.9	0.017	0.920
AEE	64.9^a^	60.8^b^	62.1^b^	61.8^b^	62.1	61.5	63.8	1.4	0.026	0.348
TDF	39.1^a^	29.8^b^	36.3^c^	32.5^b^	32.6^x^	36.4^y^	37.1^y^	2.7	< 0.001	0.022
IDF	37.6^a^	29.4^b^	35.4^c^	33.9^c^	33.4^x^	34.6^x^	35.4^y^	1.8	< 0.001	0.007
NDF	34.4^a^	26.1^b^	32.0^c^	31.6^c^	30.6^x^	31.9^x^	32.7^y^	2.1	0.004	0.031
ADF	19.1	18.5	21.8	20.8	19.3	20.9	19.9	2.4	0.231	0.611

^1^No significant interaction between dietary treatment and period (*P* > 0.10).

^2^Dietary treatments include a low-fiber control (LF) = basal corn–soybean diet; high-fiber control (HF) = basal corn-soybean diet with 30% corn bran at the expense of corn; HF with the addition of xylanase (XY); HF with the addition of arabinoxylan-oligosaccharide (AX).

^3^Pooled SEM.

^4^Dietary treatment.

^5^Apparent ileal digestibility = {100 − [100 × (% TiO_2_ in feed/% TiO_2_ in ileal digesta) × (concentration of component in ileal digesta/concentration of component in feed)]}.

^a,b,c^Within a row, means without a common superscript differ for the effect of dietary treatment (*P ≤ *0.05).

^x,y^Within a row, means without a common superscript differ for the effect of period (*P* ≤ 0.05).

**Table 4.** The main effects^1^ of dietary treatment and period on the AID of total non-starch polysaccharides (**T-NSP**), total arabinoxylans (**T-AX**), total cellulose (**T-CEL**), insoluble non-starch polysaccharides (**I-NSP**), insoluble arabinoxylans (**I-AX**), and insoluble cellulose (**I-CEL**).

	Dietary treatment^2^				Period			SEM^3^	Trt^4^ *P-*value	Period *P-*value
Item	LF	HF	XY	AX	1	2	3			
AID^5^, %										
T-NSP	30.4^a^	17.8^b^	23.9^c^	20.4^b^	21.3^x^	22.5^x^	25.5^y^	0.9	0.023	0.041
T-AX	20.3^a^	19.8^a^	29.9^b^	18.3^a^	21.9	22.4	22.3	2.7	0.012	0.612
T-Cel	30.9^a^	22.1^b^	25.2^b^	22.5^b^	24.2	25.1	26.2	1.3	0.044	0.092
I-NSP	33.1^a^	19.7^b^	26.4^c^	20.1^b^	23.4^x^	24.5^x^	28.2^y^	0.9	< 0.001	0.036
I-AX	23.7^a^	23.2^a^	34.3^b^	22.9^a^	25.9	26.3	27.0	1.8	0.007	0.387
I-Cel	36.8^a^	24.7^b^	27.5^b^	24.9^b^	26.2	27.4	28.6	2.1	0.025	0.095

^1^No significant interaction between dietary treatment and period (P > 0.10).

^2^Dietary treatments include a low-fiber control (LF) = basal corn-soybean diet; high-fiber control (HF) = basal corn-soybean diet with 30% corn bran at the expense of corn; HF with the addition of xylanase (XY); HF with the addition of arabinoxylan-oligosaccharide (AX).

^3^Pooled SEM.

^4^Dietary treatment.

^5^Apparent ileal digestibility = {100 – [100 × (% TiO_2_ in feed/ % TiO_2_ in ileal digesta) × (concentration of component in ileal digesta/ concentration of component in feed)]}.

^a,b,c^Within a row, means without a common superscript differ for the effect of dietary treatment (*P ≤ *0.05).

^x,y^Within a row, means without a common superscript differ for the effect of period (*P ≤ *0.05).

### Apparent Total Tract Digestibility

There was no significant interaction for dietary treatment by period for the ATTD of DM, GE, CP, AEE, TDF, IDF, NDF, and ADF (*P *> 0.10), so the main effects are presented in Table 5. Compared to LF, HF reduced the ATTD of DM by 12.5%, and xylanase partially mitigated this effect, increasing the ATTD of DM by 3.7% relative to HF (*P* < 0.001). Similarly, relative to LF, HF decreased the ATTD of GE by 12%, and xylanase partially mitigated this effect, increasing the ATTD of GE by 4.2% (*P* < 0.001). Compared to LF, HF reduced the ATTD of CP by 5.2%, and relative to HF, XY increased the ATTD of CP by 2.5% relative to HF (*P* < 0.001). The ATTD of DM, GE, and CP did not differ among periods (*P* = 0.103, *P* = 0.216, and *P* = 0.615, respectively). The ATTD of AEE was 12.8% lower in pigs fed HF, relative to LF, but pigs fed XY had 6.9% greater ATTD of AEE relative to HF (*P* = 0.033). The ATTD of AEE was lower in P2 and P3, relative to P1 (*P* = 0.041). Compared to LF, HF decreased the ATTD of IDF, TDF, and NDF by 15.7%, 16.3%, and 15.3%, respectively (*P* < 0.001). However, the addition of xylanase to HF increased the ATTD of IDF, TDF, and NDF by 10.6%, 12.7%, and 6.7%, respectively (*P* < 0.05). The ATTD of IDF and TDF tended to be greater in P2 and P3 compared to P1 (*P* = 0.081 and *P* = 0.085, respectively). Across dietary treatments, LF expressed the greatest ATTD of ADF (*P* < 0.001), but it did not differ among periods (*P *= 0.569).

**Table 5.** The main effects^1^ of dietary treatment and period on the ATTD of dry matter (**DM**), gross energy (**GE**), crude protein (**CP**), acid-hydrolyzed ether extract (**AEE**), total dietary fiber (**TDF**), insoluble dietary fiber (**IDF**), neutral detergent fiber (**NDF**), and acid detergent fiber (**ADF**)

	Dietary treatment^2^				Period			SEM^3^	Trt^4^ *P-*value	Period *P-*value
Item	LF	HF	XY	AX	1	2	3			
ATTD^5^, %										
DM	89.8^a^	78.6^b^	81.5^c^	78.3^b^	80.5	81.2	82.2	0.7	<0.001	0.103
GE	89.3^a^	78.4^b^	81.8^c^	78.7^b^	81.2	82.1	82.6	0.4	<0.001	0.216
CP	87.1^a^	82.6^b^	84.7^c^	82.1^b^	84.1	84.1	84.2	0.5	<0.001	0.615
AEE	58.4^a^	50.9^b^	54.7^c^	52.1^b^	56.9^x^	53.1^y^	52.9^y^	1.9	0.033	0.041
IDF	45.2^a^	38.1^b^	42.6^c^	37.9^b^	39.7	41.3	42.7	1.1	<0.001	0.081
TDF	50.9^a^	42.6^b^	44.4^c^	42.1^b^	44.4	45.1	46.2	1.0	<0.001	0.085
NDF	44.8^a^	37.8^b^	41.8^c^	37.4^b^	39.3	40.6	41.5	1.2	<0.001	0.102
ADF	36.9^a^	30.0^b^	32.9^b^	31.1^b^	32.7	33.2	33.8	1.7	<0.001	0.569

^1^No significant interaction between dietary treatment and period (*P* > 0.10).

^2^Dietary treatments include a low-fiber control (LF) = basal corn-soybean diet; high-fiber control (HF) = basal corn-soybean diet with 30% corn bran at the expense of corn; HF with the addition of xylanase (XY); HF with the addition of arabinoxylan-oligosaccharide (AX).

^3^Pooled SEM.

^4^Dietary treatment.

^5^Apparent total tract digestibility = {100 − [100 × (% TiO_2_ in feed/ % TiO_2_ in feces) × (concentration of component in feces/ concentration of component in feed)]}.

^a,b,c^Within a row, means without a common superscript differ for the effect of dietary treatment (*P ≤ *0.05).

^x,y^Within a row, means without a common superscript differ for the effect of period (*P* ≤ 0.05).

## Discussion

It is well-established that fiber is not well utilized by pigs; it commonly exerts an antinutritive effect that results in decreased nutrient and energy digestibility and often reduced growth performance (Agyekum and Nyachoti, 2017). The majority of fiber native to corn grain and its co-products is highly insoluble and scantily fermented by resident microbiota (De Vries et al., 2012; Gutierrez et al., 2014). This is apparent herein as a 30% addition of corn bran without solubles, an industrial co-product high in IDF, decreased the digestibility of nutrients and energy across the gastrointestinal tract. The decreased AID and ATTD of nutrients and energy observed in HF, compared to LF, agrees with other studies evaluating the digestibility of corn and its co-products that are rich in IDF (Gutierrez et al., 2013; Acosta et al., 2020). The decrease in ATTD of nutrients observed in HF is greater than what can be explained by the digestibility of individual ingredients suggesting that IDF decreases the digestibility of other nutrients within the diet matrix. This decrease is likely a product of the increased arabinoxylan content in corn bran; corn-based arabinoxylans are poorly fermented by gastrointestinal microbiota due to their abundance of L-arabinofuranosyl substitutions, lignin cross-bridges, and subsequent insolubility (Bach Knudsen, 2014). Moreover, the concentration of arabinoxylan among 9 corn co-products best explained the variance in the AID of GE and DM in growing pigs (Gutierrez et al., 2014). Likewise, xylose, a constituent of arabinoxylan, explained most of the variability observed among the ATTD of GE, DM, and NDF within the same study.

Both the indigestible nature and abundant concentration of arabinoxylans found in corn and its co-products provide a logical motive for supplementing xylanase in swine diets. However, the efficacy of xylanase in swine production is highly variable, and there is a poor understanding of why it appears to be less effective in corn-based diets relative to other arabinoxylan-rich cereal grains and compared to poultry studies (Torres-Pitarch et al., 2019; Petry and Patience, 2020). One experimental condition that appears to affect the ability of xylanase to improve the growth performance of pigs fed corn-based diets is the length of supplementation time (Myers and Patience, 2014; Lan et al., 2017; Petry et al., 2020a). There is a paucity of studies directly investigating the impact of adaptation time on the efficacy of xylanase. The aim of this experiment was to evaluate the impact of xylanase on viscosity and nutrient and energy digestibility over time in multiple gastrointestinal locations of growing pigs fed a diet higher in corn-based fiber.

Arabino-xylooligosaccharides are thought to be one release product from the hydrolysis of arabinoxylans by xylanase (Pedersen et al., 2015; Ravn et al., 2016); in poultry, directly supplementing them has been shown to elicit similar responses to xylanase addition to the diet (Morgan et al., 2019; Bautil et al., 2020). Therefore, it was also of interest to investigate the impact of directly supplementing AXOS over time in the pig. Directly supplementing AXOS appeared to improve the AJD of digestibility of fiber, DM, and GE in P1 and P2, but not in P3. The transient response of AX in the upper small intestine is challenging to explain, but it is plausible that it is due to the interactive effects of AXOS on small intestine microbiota. The proposed mechanism of action for AXOS is that it improves fiber digestibility by stimulating the growth of intestinal microbiota that ferment arabinoxylan more efficiently (Bedford, 2018; Bautil et al., 2020). Perhaps this microbial modulation is not sustainable in the jejunum or conceivably, the benefit of AXOS moves aborally with age. Another explanation could be that fermentable substrates concentrate as they progress towards ileal indigesta in swine (Moran and Bedford, 2022). This might explain why AX improves the AID of IDF and TDF over HF, irrespective of adaptation time. Further research is warranted to better understand the transient effect of AX supplementation and if it exerts a similar mechanism of action within the pig’s intestine as it does in poultry.

The interaction between dietary treatment and period for the AJD of DM, GE, TDF, NDF, and IDF observed herein indicates that the efficacy of xylanase to improve fiber digestibility in the upper small intestine increases with a greater adaptation time. The impact of adaption time on the efficacy of xylanase to improve AJD of DM, GE, and fiber, but not AID and ATTD, may partially explain why digestibility responses are more common within the literature than performance responses (Petry and Patience, 2020). The adaptation period implemented in digestibility studies is frequently less than 10 d; in this study, xylanase improved the AID and ATTD of NDF, TDF, and IDF after 7 and 10 d of adaptation, respectively. In contrast, it took 25 d of adaptation for xylanase to yield an AJD response. In theory, if increasing supplementation time shifts the action of xylanase into the small intestine, it may improve the absorption of monosaccharide-release products. This may increase the energy available to the pig as energy derived from carbohydrate fermentation is metabolically less efficient when compared to direct absorption and utilization of carbohydrates. Moreover, the fermentation of fiber is energetically expensive which may increase metabolic heat production, and thus, the maintenance energy requirement of the pig (Noblet and Le Goff, 2001; Agyekum and Nyachoti, 2017). However, it should be noted that it is unclear if the metabolic efficiency of xylose is greater when fermented to a short-chain fatty acid or metabolized by the pig as a monosaccharide (Verstegen et al.,1997; Huntley and Patience, 2018). Furthermore, it is ambiguous if xylanase can release individual monosaccharides in vivo from corn-based arabinoxylans (Dale et al., 2019). Additional research is needed to determine the composition of release products from the enzymatic action of xylanase in the upper small intestine, and the metabolic efficiency by which the pig uses them for energy.

The increased AID of DM, GE, NDF, TDF, and IDF observed in XY are in agreement with Passos et al., (2015) who discerned similar results in growing pigs fed a corn-based diet supplemented with xylanase. Likewise, Chen et al., (2020) reported xylanase improved the AID of DM, GE, and NDF, but not ADF in nursery pigs fed a diet with a 30% inclusion of corn distiller’s dried grains with solubles (**DDGS**). The improvements in ATTD of DM, GE, and TDF also align with a study evaluating the impact of xylanase supplementation with corn samples varying in their NDF and ME content (Petry et al., 2019). Conversely, Abelilla and Stein (2019) supplemented two sources of xylanase, different from the enzyme utilized herein, in both corn-based and corn DDGS-based diets, and neither improved the apparent duodenal digestibility, AID, and ATTD of similar dietary components.

There are three commonly proposed mechanisms by which xylanase could improve nutrient and energy digestibility. First, it is reasonable to suggest xylanase improves the digestibility of fiber through the hydrolysis of arabinoxylan into absorbable components (i.e., monosaccharides), or fermentable fragments such as AXOS and low molecular-weight polysaccharides (Adeola and Cowieson, 2011). This mode of action would certainly support the AJD and AID of AX, NSP, TDF, IDF, and NDF observed herein. Moreover, it would provide a more fermentable substrate for hindgut microbiota which would support the increased ATTD of DM, GE, TDF, IDF, and NDF in XY, compared to HF.

**Figure 7.**
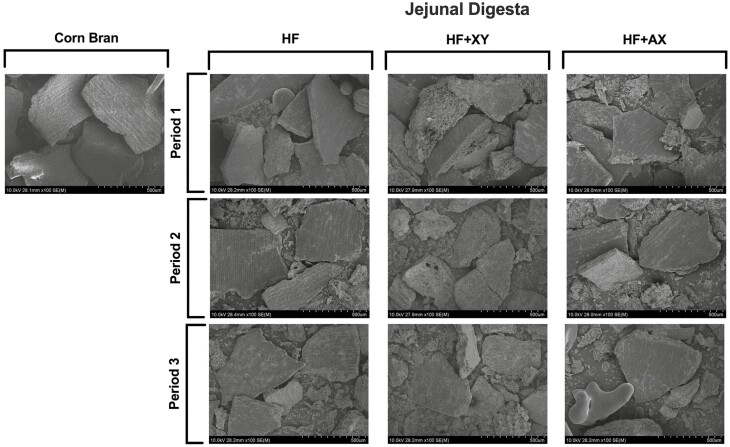
Field emission scanning electron microscopy images (100×) of corn bran, and jejunal digesta from pigs fed high-fiber dietary treatment (**HF**), HF plus xylanase (**XY**), and HF plus arabinoxylan oligosaccharide (**AX**) in P1, P2, and P3. Representative images were selected by a research technician blinded to treatments.

**Figure 8.**
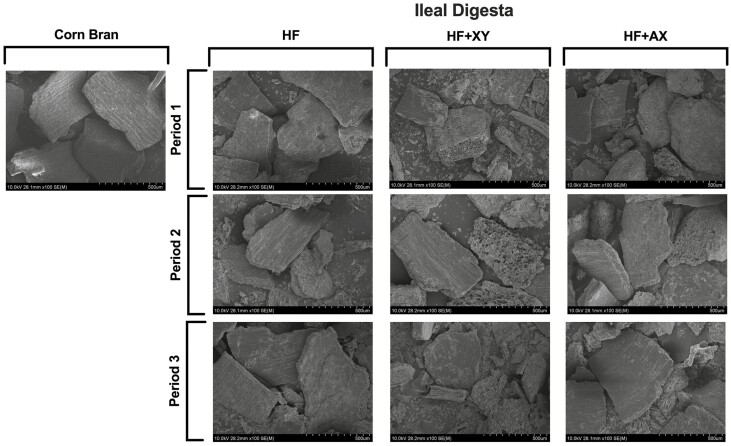
Field emission scanning electron microscopy images (100×) of corn bran, and ileal digesta from pigs fed the high-fiber dietary treatment (**HF**), HF plus xylanase (**XY**), and HF plus arabinoxylan oligosaccharide (**AX**) in periods 1, 2 and 3 (P1, P2, and P3). Representative images were selected by a research technician blinded to treatments.

Second, supplementing xylanase may partially mitigate the nutrient encapsulation effect of fiber by exposing nutrients that are otherwise trapped within the plant cell and inaccessible to either endogenous enzymes in the small intestine or microbes in the hindgut (de Lange et al., 2010). Field emission SEM was used herein to evaluate the topography of corn bran particles identified in the jejunal and ileal digesta from pigs fed HF, XY, and AX (Figs. 7 and 8, respectively). The representative SEM images of ileal digesta of XY from P2 and P3 provide some visual, but unquantifiable, evidence that xylanase disrupts the pericarp structure of bran and may expose attached vitreous endosperm to endogenous enzymes. When images of ileal digesta in P2 and P3 are compared to SEM images of corn bran, it appears more of the underlying vitreous endosperm are exposed and particles have greater surface area in the XY treatment. However, the SEM images of jejunal digesta provide little intelligible evidence of this occurring in the upper small intestine. The improved accessibility of trapped lipid and protein components would also support the hypothesis of improved ATTD of CP and AEE by xylanase. On the other hand, these responses could be a result of increased microbial fermentation of CP and AEE as xylanase and its release products can modulate a more metabolically active microbiome (González-Ortiz et al., 2019).

Third, xylanase may attenuate the physicochemical properties of fiber that negatively impact nutrient digestibility, such as increased digesta viscosity (de Vries et al., 2012). The success of xylanase in poultry production has been attributed to its efficacy in ameliorating increased digesta viscosity (Raza et al., 2019), but this phenomenon is variable in swine diets (Patience et al., 1992; Laerke et al., 2015). Indeed, neither dietary treatment nor period affected jejunal or ileal viscosity measurements herein. This is not surprising as corn and its co-products do not form viscous gels, and thus, have a limited impact on digesta viscosity (Navarro et al., 2018). However, this is in opposition to other studies evaluating the viscosity of jejunal digesta from pigs fed corn-based diets (Passos et al., 2015; Tiwari et al., 2018; Chen et al., 2020). These conflicting results are likely a product of differing methodologies as these studies measured the viscosity of digesta supernatant at a limited number of shear rates. In contrast, herein the viscosity of whole digesta was measured because the supernatant is not directly indicative of the rheological properties of whole digesta (Takahasi and Sakata, 2004). Digesta also displays pseudoplastic shear-thinning behavior in that when the shear rate of the viscometer increases, viscosity decreases (Dikeman and Fahey, 2006). Consequently, when viscosity is measured at one or two shear rates it may inadequately depict the profile of rheological properties of digesta.

## Conclusions

In conclusion, as expected, increasing TDF through the inclusion of corn bran without solubles decreased nutrient and energy digestibility across the gastrointestinal tract irrespective of adaptation time. Supplementing AXOS to HF resulted in a transient improvement in the AJD of nutrients and energy, in that digestibility was improved in P1 and P2, but not P3. In contrast, as adaptation time increased, XY improved the AJD of nutrients and energy relative to a high-fiber control. The impact of adaptation time on the efficacy of xylanase’s ability to improve fiber digestibility in the upper small intestine may partially explain why increased adaptation time seems to improve growth performance. However, irrespective of adaptation time, xylanase improved the AID and ATTD of TDF, NDF, and IDF. This is likely the result of xylanase hydrolyzing arabinoxylan into more digestible or fermentable components, and not a result of attenuating digesta viscosity. Moreover, SEM images of ileal digesta and the improved ATTD of AEE and CP by xylanase support the notion that this enzyme partially ameliorates the nutrient encapsulation effect of fiber. Holistically, these data imply that supplementing xylanase over several dietary phases may improve its efficacy in pork production.

## Abbreviations

ADF: acid detergent fiber
AEE: acid-hydrolyzed ether extract
AJD: apparent jejunal digestibility
AID: apparent ileal digestibility
ATTD: apparent total tract digestibility
AX: arabinoxylan-oligosaccharide diet
AXOS: arabinoxylan-oligosaccharide
CP: crude protein
DDGS: distiller’s dried grains with solubles
DM: dry matter
GE: gross energy
HF: high-fiber control
IDF: insoluble dietary fiber
I-AX: insoluble arabinoxylans
I-CEL: insoluble cellulose
I-NSP: insoluble non-starch polysaccharides
LF: low-fiber control
NDF: neutral detergent fiber
NSP: non-starch polysaccharides
SDF: soluble dietary fiber
SID: standard ileal digestible
STTD: standardized total tract digestible
TSAA: total sulfur amino acids (Met + Cys)
T-AX: total arabinoxylans
T-CEL: total cellulose
T-NSP: total non-starch polysaccharides
TDF: total dietary fiber
XY: xylanase diet
